# Using World Cafés to engage an Australian culturally and linguistically diverse community around human papillomavirus vaccination

**DOI:** 10.1111/hex.13703

**Published:** 2023-02-16

**Authors:** Kathleen Prokopovich, Lyn Phillipson, Leissa West (Pitts), Biljana Stanoevska, Jackie Street, Annette Braunack‐Mayer

**Affiliations:** ^1^ Australian Centre for Health Engagement, Evidence and Values, School of Health and Society, Faculty of the Arts, Social Science and Humanities University of Wollongong Wollongong New South Wales Australia; ^2^ School of Health and Society, Faculty of the Arts, Social Science and Humanities University of Wollongong Wollongong New South Wales Australia; ^3^ Multicultural and Refugee Health Service Illawarra Shoalhaven Local Health District Warrawong New South Wales Australia; ^4^ School of Public Health, Faculty of Health and Medical Sciences The University of Adelaide Adelaide South Australia Australia

**Keywords:** cultural diversity, human papillomavirus, participatory, school delivery, trust, vaccination

## Abstract

**Introduction:**

Internationally, cultural factors are associated with vaccine uptake and completion in ethnic minority communities. Whilst Australia has achieved high human papillomavirus (HPV) vaccination, little is known about how culture or ethnicity influences HPV vaccination engagement. To address these gaps, we partnered with our Local Health District to explore how one culturally and linguistically diverse (CALD) community engages with school and HPV vaccination.

**Methods:**

We adapted a participatory research method (the World Café) to engage one local CALD community—the Macedonian community (Our bi‐cultural researcher and participants preferred the term ‘Macedonia’ rather than The Republic of North Macedonia as outlined in the 2018 Prespa agreement) in New South Wales (Australia)—to discuss HPV and school vaccination. Our qualitative analysis combined deductive codes taken from the Tailoring Immunization Programme framework, inductive codes guided by narrative inquiry (temporality, sociality and place) and previously known vaccination ‘trust’ frameworks.

**Results:**

In late 2019, 31 local Macedonian community members were purposely recruited for two World Cafés (*n* = 15 mothers/grandmothers and *n* = 16 young adults). Our themes reveal a community narrative grounded in historical vaccine experiences, family views on vaccination and a general trust in schools. Participants collectively discussed how ‘increasing knowledge’ and ‘tailoring health communications’ could strengthen community vaccine decision‐making.

**Conclusion:**

This study demonstrates how research partnerships and participatory methods can be applied in CALD community settings to research engagement with school and HPV vaccination. Our World Café dialogues highlight a positive narrative about vaccines, where community vaccination behaviours were built on multilayer trust relationships despite low vaccine knowledge. Our findings further knowledge around ‘public trust’ in school vaccination, highlighting the importance of existing (or missing) trust relationships when tailoring vaccine communication to local CALD communities.

**Patient or Public Contribution:**

Participants who took part in the World Cafes were all local Macedonian community parents or young adults who have been or will be exposed to the health services offered by school‐based HPV vaccination. Thus, all the data collected came from their personal experiences with the school vaccination programme, or how they expect to participate in the programme. To ensure our study design was culturally appropriate and tailored to the Macedonian community, we engaged with the relevant local health stakeholders (the bi‐cultural Multicultural Health Officer and Multicultural Health Service Manager Programme Director) to adapt and refine the World Café method for this context and setting. Our local health stakeholders also reviewed our preliminary findings, assisted with data interpretation and participated in manuscript editing.

## 1 INTRODUCTION

The elimination of cervical cancer by 2030 is dependent on high coverage of human papillomavirus (HPV) vaccination, cervical screening and treatments of cervical lesions.^1^ Even when countries such as Australia, New Zealand and Latin America are achieving high national HPV vaccination coverage,^2^ challenges remain to ensure equitable vaccination and screening in all population subgroups.^3^ In many countries, compared to the mainstream population, those from racial/ethnic or culturally and linguistically diverse (CALD) communities are less likely to complete HPV vaccination and participate less in cervical screening programmes.^4^, ^5^, ^6^, ^7^, ^8^ For CALD groups, barriers to accessing cervical cancer prevention programmes related to mainstream language proficiency; first‐ or second‐generation status; access to healthcare services and trust in vaccine information and safety.^9^, ^10^ As health authorities generally aim to administer HPV vaccination before adolescents are 15 years old, some CALD parents may be hesitant to consent to vaccination due to perceptions that the HPV vaccine could encourage earlier sexual activity.^11^

However, CALD communities are heterogeneous and must be untangled from each other to understand their unique vaccination contexts and perspectives.^10^ For example, international research shows that CALD parents may turn to interpersonal networks when seeking HPV vaccination advice.^12^, ^13^, ^14^, ^15^ Depending on the community, this advice could occur mother to mother (as seen in some Korean–American communities)^14^ whilst African mothers may defer decision‐making to fathers.^15^ These variations demonstrate a need for place‐based research and engagement with individual CALD communities, so vaccination interventions can be tailored appropriately.^16^

In Australia, the national school‐based HPV vaccination programme began in 2007, delivering the vaccine to adolescents 12–14 years old. Since then, a significant reduction in HPV disease has been observed^17^, ^18^, ^19^, ^20^ and national coverage is now about 82.6% for females and 79.9% for males.^20^ In spite of these national gains, local‐level data reveals variable vaccine coverage based on gender, socioeconomic status and geographical location.^20^, ^21^, ^22^, ^23^ However, as CALD data are not collected in vaccination registers, assessing if any CALD communities experience vaccination coverage inequities is difficult. To date, only two Australian studies have reported vaccination coverage for students with ‘a language background other than English’ with neither study finding an association.^21^, ^23^ However, these studies did not specify the language backgrounds included. With some immunization providers reporting much lower vaccination coverage of around 53.2% in schools or communities with higher CALD representation,^20^ it is possible that as seen in Canada^24^ and England,^25^ associations between lower vaccination coverage and CALD status are being masked.

Consistent with previous findings that available Australian vaccination information is ‘difficult to read’,^26^ Australian immunization providers have reported that language barriers and health literacy negatively influence the success of the school vaccination programme.^20^ There is also limited Australian literature describing place‐based research activities with priority CALD communities to identify factors that facilitate or impede engagement with school vaccination. To address these knowledge gaps, in 2019, researchers from the University of Wollongong and the Illawarra‐Shoalhaven Local Health District (ISLHD) partnered to take part in a participatory action research project which adapted a participatory research method, the World Café^27^, ^28^ to engage one local CALD community in dialogue around HPV and school vaccination, and highlight where the programme could be improved for their community.

World Cafés (herein called Cafés) have been used successfully in other multicultural Australian settings to promote two‐way dialogue,^29^ and draw on seven (adaptable) design principles to host ‘conversations that matter’ and create ‘innovative possibilities for action’.^27^, ^28^ In keeping with other strength‐based approaches in minority cultural settings,^30^ Cafés provide a psychologically secure setting, promote diverse ways of thinking and form deeper collective understandings of the topics being discussed. ^27^, ^28^ Details of our Café approach are found in Table 1 and Supporting Information.

**Table 1:** Adapting the principles for World Cafés to the Macedonian community

World Café principles^27^	Description taken from Brown and Isaacs^27^	How applied in this research
Context	Three contextual elements include having:	
	(1) Clear purpose for the Café.	(1) Clear research aims were established by reviewing current literature and discussing shared goals with our ISLHD partners.
	(2) A target of participants for the Café.	(2) Target Population—Macedonian community members (to ensure cultural safety only these Cafés are restricted to one CALD community, diversity will be achieved by participant age or gender).
	(3) Clear parameters for the World Café.	(3) Clear budget and research timeline.
Create a hospitable space	Ensures that all participants and the research team feel welcome, safe and psychologically secure.	Research location was chosen by our ISLHD partners and known to local participants. Checked tablecloths were used, and there was unlimited access to coffee, tea, fruit and sandwiches. We consulted with our ISLHD partners about all the details.
Explore questions that matter	World Café questions facilitate dialogue around strategic questions which promote collaborative engagement.	The questions asked in these World Cafes were guided by the aims and objectives of the research topic, the research team's previous experiences with the World Café method and discussions with the local ISLHD. All research team members reviewed and approved the questions (Box 1).
Encourage everyone's contribution	All participants will be encouraged to be involved with the community dialogue by the research team emphasizing that everyone has a contribution that is worth sharing.	Each table had a research team member acting as a ‘host’ to scribe and guide the conversation. Hosts ensured everyone had a chance to speak. Language assistance was offered by our bicultural research team member.
Connect diverse perspectives	Table covers are available for participants to reflect and build on what has been discussed during the previous table conversations.	Brown table paper, coloured markers and pens were available for participants and research team members to write on. Research ‘hosts’ reviewed all previously scribed points to participants as they shuffled from table to table.
Listen together for patterns and insights	Participants will be prompted to use shared listening and pay attention to all the perspectives discussed.	Participants were encouraged to look at the table papers and highlight what they agreed or disagreed with and were prompted to share their experiences.
Share collective discoveries	Opportunity to reflect as a large group on the ideas, perspectives and issues shared throughout the process.	During the last break period, the scribed table paper was brought to the lead researcher and each paper was summarized onto a PowerPoint slide. These slides were presented and reviewed by all participants. Participants were provided an opportunity to share and reflect on the table answers and insights.

Abbreviations: CALD, culturally and linguistically diverse; ISLHD, Illawarra‐Shoalhaven Local Health District.

### 1.1 The Wollongong and Shellharbour Macedonian community

Currently, the Macedonian community in Wollongong and Shellharbour (NSW, Australia) is one of the largest established non‐English speaking ancestries in the area (3.23% and 3.24%, respectively, compared to the Australian total population of 0.4%)^32^ and the Macedonian language is ranked first as a language spoken at home after English (2.2%–2.3% compared to 0.3% of the Australian population).^32^, ^33^ Macedonians in NSW also report more difficulty in speaking English compared to the State average (11.9%–4.5% respectively),^34^ and are a community the ISLHD identifies as a CALD population group requiring additional support to engage with preventative health services.^35^ As we had little knowledge of this community's HPV vaccination coverage, our health district research partners chose the Macedonian community as a priority group for this engagement research.

Macedonian migration to Australia has occurred in four waves since 1920. The largest migration occurred in the 1960s when many Macedonians left the Socialist Republic of Yugoslavia.^36^ At that time, the NSW steel industry employed many migrant Macedonian men, and when the industry relocated to Wollongong, the men and their families followed.^37^ Typically, community members moved to Wollongong and Shellharbour suburbs with others from the same Macedonian village^37^, ^38^ allowing neighbours to unite together to celebrate traditional village life.^38^ While the husbands worked, wives stayed home to raise children thus many missed out on education in Australia (Personal communication, Bicultural worker email). Currently Macedonian's in NSW have a larger percentage of persons with ‘No qualifications’ as compared to the NSW State average (55.9% compared to 39.1%, respectively) and a smaller percentage of persons with Bachelor or Higher degrees (13.5% compared to 23.4% of NSW).^39^ Approximately, 8808 Macedonians^32^ still live in the area, celebrating their culture through dancing groups, soccer clubs, Saturday language schools, religious services and Macedonian Welfare Association‐sponsored events.^40^

### 1.2 Aims and objectives

This study incorporates a more ‘northern tradition’ of participatory research, where the driver of partnership engagement was to understand factors which facilitate or impede health programme (i.e., school‐based vaccination) use and identify areas where our research partnership could improve community use of the programme.^41^ To accomplish this, our academic‐community stakeholder partnership aimed to answer the following question:

How does an established cultural and linguistically diverse (CALD) community perceive and engage with their local school vaccination program and HPV vaccination?

## 2 METHODS

### 2.1 Participatory research and the World Café

To foster constructive, culturally safe Café dialogues, we actively engaged with our ISLHD research partners, a local Macedonian bicultural Health Officer (B. S.) and Multicultural and Refugee and Migrant Health Service Manager (L. W. (P.)), to adapt Café principles to the local Macedonian community context (Table 1). Standard principles for Cafés include: clarifying the context; creating a hospitable space; exploring questions that matter; encouraging everyone's contribution; connecting diverse perspectives; listening for patterns and insights and sharing collective discoveries. These design principles allow participants to be ‘experts of their own lived experiences’, produce local knowledge and identify potential solutions.^27^ On the advice of our ISLHD partners, our adaptations to the usual Café method included limiting diversity to one community and asking participants their preferred way to achieve diversity within Cafés (i.e., by age range or by gender). As information sharing is part of the trust‐building the ISLHD performs to reach communities, the Manager (L. W. (P.)) also requested that we present participants with basic information on HPV and school vaccination. We agreed to present this information before Cafés started. This provided health education to participants and ensured participants could confidently engage on Café topics and the language of vaccination.

### 2.2 Recruitment

Using the preferred CALD consumer recruitment method of our ISLHD partners,^42^ our bicultural research team member (B. S.) approached all potential participants face‐to‐face at established Macedonian dancing and meeting groups. Whilst this bicultural officer is known to the local Macedonian community, she is not in any dependent relationships with community members. To ensure a diverse range of community perspectives in each Café, we aimed to recruit mixed‐gender groups with each Café hosting a minimum of 12 participants (i.e., at least 4 people per table). We purposely recruited: (i) parents or grandparents of a child/grandchild aged 7–24 years old (those who have consented or will be approached to consent for school vaccination); and (ii) young adults aged 18–24 years old who had been exposed to their local school vaccination programme. This allowed participants to speak from either their own past experiences or how they hope to experience the programme. Eligible participants could participate if they spoke English and could read and understand the English research information sheet. Participants who verbally consented to participate were consulted about the best location, date and time for the two discussion groups. Before Cafés, B. S. called potential participants to confirm attendance, provide venue information and answer questions. At Cafés, participants were offered light refreshments and a shopping voucher as reimbursement for time and effort to attend.

### 2.3 World Café method

To promote a well‐rounded discussion about HPV and school vaccination the lead author (K. P.) and two senior researchers (J. S. and A. B.‐M.—both with expertise in public deliberation, bioethics and conducting Cafés) developed table questions (Box 1). Proposed questions were presented to the research team, and after discussion and consultation, all research team members approved the questions.

Box 1. **School‐based vaccination World Café table questions**

**Table:** 

World Café table question(s)
Table 1: ‘What do you think about high school students receiving vaccinations at school? What does your community think?’
Table 2: ‘What do you think about the human papillomavirus vaccination? What do members of your community think?’
Table 3: ‘For you and other people from your community, what is the best way to give information about and get consent for the human papillomavirus vaccination?’
All Tables: ‘From your perspective, if the high school vaccination program was run differently, what would make it better for you and your community members?’

Cafés lasted 95 min and were held on a weekday evening in late 2019. Held at a function centre familiar to participants, parents/grandparents attended the first Café and young adults the second. The room had three round tables which could sit up to eight people. Table facilitators were all trained and experienced in qualitative methods and included two authors (L. P. and J. S., with L. P. having previous experience facilitating focus groups with Macedonian community members) and a third public health researcher. The programme for Cafés can be found in Supporting Information. Facilitators had a discussion guide (available in Supporting Information), large sheets of table paper and markers to scribe and highlight discussion points (‘collective discoveries’) on the table paper. The lead author (K. P.) coordinated and hosted the event. Our ISLHD research partners (L. W. (P.) and B. S.) and an ISLHD administrative support person also attended.

Upon arrival, participants sat at a table of their choosing and provided informed consent by reading and signing an English language consent form and completing a short demographic survey. Language support was available through B. S. upon request. K. P. informed everyone that discussions would be digitally recorded and participants could withdraw at any point. Participants were asked not to share any personal stories they heard outside of the Cafés.

The lead author presented information about the research project and information on HPV and school vaccination (available in Supporting Information). Participants were asked a warm‐up question, and after discussing answers as a group, facilitators then guided the discussion of the assigned table question (Box 1). Up to 15 min were given to answer each table question. While participants talked, facilitators scribed discussion points on the table paper (example image provided in Supporting Information). Before answering a new question, participants were asked to move to a new table and sit with new people. This format continued until all four questions were answered. During the last break, all facilitators summarized the ‘collective discoveries’ on the table paper and presented these to the lead author. After the break, the lead author presented all scribed table paper ‘discoveries’ to participants, and participants were given an opportunity to confirm, disagree or elaborate on the findings.

After the Café events, K. P. and B. S. made reflective field notes. Scribed table paper was photographed for data analysis, and digital audio recordings of the English language discussions were transcribed verbatim and de‐identified by K. P.

### 2.4 Data analysis

Transcripts and table paper images were imported into NVIVO software (version 12.6.1). K. P. conducted the initial analysis of each transcript using both deductive and inductive coding.^43^ Deductive codes were taken from the Tailoring Immunization Programme (TIP) theoretical framework which categorizes childhood immunization behavioural factors related to capability; motivation; physical opportunity and social opportunity.^44^ Identified codes and themes not sitting within the TIP framework were added to the codebook, and previous transcripts were reviewed and recoded. K. P. conferred with L. P. and A. B.‐M. throughout the process to review, refine and develop key conceptual (latent) codes and themes. During these talks, L. P. and A. B.‐M. drew and reflected on their previous research experiences with HPV vaccination (A. B.‐M.) and CALD community barriers to cancer prevention information (L. P.). Any discrepancies were discussed until a consensus was reached. Our bicultural health officer (B. S.) reviewed the initial results and provided cultural context and interpretations of the themes. A sample of this analysis is available in Table 2 and Supporting Information (Sup Mat_1).

**Table 2:** Example of deductive and inductive themes for data analysis

Theme	Subthemes	Sample quotes
We've Always Vaccinated	*Deductive (TIP framework)*	‘I think when [parents] first have their kids they [have] an understanding what vaccinations are—they can get it from 6 months, 4 years, it's something that is always done for their kids. So, I guess looking into the understanding of what these new vaccinations are they don't really care necessarily. They just know it's going to help their children’. (Young Adults Table 1: Second Question Period)
	Vaccine knowledge (capability).	
	Vaccines protect/help (motivation).	
	Children always vaccinated (social opportunity and motivation).	
	*Inductive (narrative inquiry)*	
	Lack of motivation to understand vaccine information (social and emotional context of vaccination).	
	Relationship of past experiences to future decisions (temporality).	
We Trust the Schools	*Deductive (TIP framework)*	‘It's a must, it's a must, it's from school, so it's a must’. (Parents Table 2: Third Question Round) ‘[Parents] think it's safe because its coming from the school’. (Young Adults Table 1: Third Question Round)
	Vaccine acceptance (motivation).	
	Vaccination attitudes (motivation).	
	Accessing vaccines (opportunity—physical).	
	Parents normally follow school recommendations (opportunity—social).	
	*Inductive (narrative inquiry)*	
	Schools as a location (place).	
	Schools are safe (social and emotional context of vaccination).	
Family Views are Passed Down	*Deductive (TIP framework)*	‘And not just speak to the kids about it because like the parents is where we trust, and like even our grandparents, we absorb so much information from them and knowledge that—if they don't trust it—it comes down to us and we're like “Well if our parents don't trust it and our family doesn't trust it why would we trust it?” You know what I mean?’ (Young Adults Table 1: Fourth Question Round)
	Knowledge (capability)	
	Family support (opportunity—social).	
	Vaccine attitudes/beliefs (motivation).	
	*Inductive (narrative inquiry)*	
	Relationship between family vaccine attitudes (motivations) and knowledge (capability) to individual motivations (social and emotional context of vaccination).	
	Family influence (social opportunity) on who/what to trust (motivations) (social and emotional context of vaccination).	
Impacts of Increasing HPV Vaccination Knowledge	*Deductive (TIP framework)*	‘But it's still like—if they [parents] knew what it [the vaccine] was they would be much more resistant I think to it’. (Young Adult Table 1: Third Question Round) ‘And I know we've heard what meningococcal is, but we don't know exactly what happens if you get it. Sit us down, slide shows, all that. That would be helpful’. (Table 2, First Question Round)
	Vaccine knowledge (capability).	
	Access/understanding vaccine information (opportunity—physical).	
	*Inductive (narrative inquiry)*	
	Child's perception of parents (social and emotional context of vaccination).	
	Relationship between knowledge (capability) and perceived risk of disease (motivations) (social and emotional context of vaccination).	
Tailored Vaccination Communication	*Deductive (TIP framework)*	*Parent 1*: ‘Ours is called a [App Name]. So, everything goes into that School Bag App and now it's called [App Name]’. *Researcher*: ‘And that tells you more information?’ *Parent 1*: ‘Yeah, that tells me everything’. (Parent, Table 3: First Question Round) ‘I can understand it. But some things I can't. That's why I always pass them piece of paper or the information to my husband to be [clear]…. Like I know how to read but some words like—I'm thinking—geez what's this word. I have no idea’. (Parent, Table 1: Second Question Round)
	Vaccine knowledge (capability).	
	Understandable information (capability and opportunity—physical).	
	Communication methods (opportunity—physical).	
	*Inductive (Narrative Inquiry)*	
	Relationship between vaccine literacy (capability), vaccine confidence (motivation) and preferred communication methods (opportunity—physical) (social and emotional context of vaccination).	

Abbreviations: HPV, human papillomavirus; TIP, Tailoring Immunization Programme.

As analysis progressed, an overarching community narrative linking the importance of schools (a place), that experienced historic vaccination events and their influence on decision‐making and trust relationships between families and institutions formed across all transcripts. As some themes stood outside of the TIP framework, K. P. compared the developing community narrative to other published social science methodologies. K. P. saw the community narrative mapping naturally to the dimensions of narrative inquiry (temporality‐events having a past, present and future; sociality‐attention to individual emotions/motivations as well as the social context and place‐physical location of the situation and its impact on inquiry)^45^ and this assisted with refining the analysis (Table 2 and Supporting Information). To explore and further conceptualize the community trust relationships, K. P. again turned to published literature on multilevel vaccination frameworks.^46^, ^47^ These results will be discussed further in Section 4.

## 3 RESULTS

### 3.1 Participants

All participants lived in Wollongong and Shellharbour (NSW), and demographics are presented in Table 3. The total number of potential participants approached was not recorded, but 16 parents and 17 young adults initially expressed interest in attending the Cafés. During reminder calls, one male parent and one young adult dropped out without providing a reason.

**Table 3:** Demographics of World Café participants

Demographic characteristics	Group 1: Grandparents/parents (*n* = 15)	Group 2: Young adults (*n* = 16)
Male	0	8
Female	15	8
Age range		
18–24	‐	16
25–45	5	‐
46–65	6	‐
66+	4	‐
Child or grandchild age range^a^		Not applicable
7–14	5	
15–24	9	
Age not identified	5	
Do you speak Macedonian as a second language at home?	Yes = 14	Yes = 13
	No = 0	No = 3
	No response = 1	No response = 0
Has your child(ren) or grandchild(ren) had the HPV vaccination?^a^	Yes = 9	Not applicable
	No = 3	
	I don't know = 4	
If you do not know, if offered today, would you consent to your child(ren) or grandchild(ren) having/have the HPV vaccination?	Yes = 4	Not applicable
	No = 0	
	Maybe = 1	
	No response = 10	

Abbreviation: HPV, human papillomavirus.

^a^ Multiple answers allowed.

### 3.2 The community vaccine narrative

**Figure 1:**
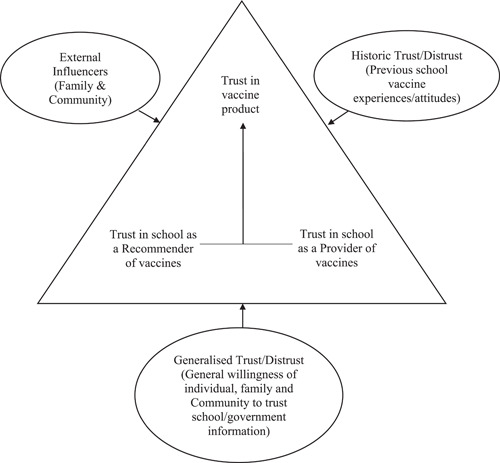
One culturally and linguistically diverse community's school vaccination trust relationship. *Source*: Larson et al.^46^

Participants drew on personal or family examples and experiences when responding to questions. These shared stories revealed an overarching ‘Macedonian vaccination narrative’ where participants identified as a community that: ‘was vaccinated’; ‘trusts’ the schools and ‘passes on’ views to their family members. These themes contextualized how ‘increasing knowledge’ and ‘tailored health communications’ could strengthen community vaccine decision‐making. Our findings demonstrate a multilayer trust relationship which is conceptualized in Figure 1.

#### 3.2.1 We've Always Vaccinated

Irrespective of age or where they grew up, most participants remembered and were familiar with receiving vaccines through schools. Many thought ‘the majority [of Macedonians] are accepting of vaccinations’ for children and school vaccines were seen as an extension of earlier childhood vaccine programmes.

I think when [parents] first have their kids they [have] an understanding what vaccinations are—they can get it from 6 months, 4 years, it's something that is always done for their kids. So, I guess looking into the understanding of what these new vaccinations are they don't really care necessarily. They just know it's going to help their children. (Young Adults Table 1: Second Question Round)

Those who grew up in Macedonia recalled being vaccinated by nurses who ‘…would come a few times a year [to school] … and then you could see the little scars’ on everyone's arm. Participants labelled this ‘the European scar’ and used it to identify those who could be European‐born.

Participants also shared stories about the benefits of school vaccination in Australia. One young adult shared how her grandparents, like many community members in the 60s and 70s, ‘…didn't know how to speak English’. Consequently, this limited their access to health information, including knowing what vaccines older children might need. She thought school vaccination programmes provided needed health care and overcame typical health communication barriers faced by migrants.

For a few in the parents/grandparent's discussion group (hereafter referred as the parent group), they remembered how their parents (the first generation of migrants) seemed unaware that children were vaccinated at school. After receiving a school vaccine, one parent remembered being asked, ‘…and what was that for?’. Regardless of the lack of knowledge surrounding vaccines, the majority of participants recalled always being vaccinated as children. ‘In her day’, one parent remembered school vaccines were painful, but her parents made her have them. Young adults also said they were ‘told to do it’ by their parents, with one young adult recalling how his mother ‘lost it’ when he purposely missed a school vaccine. He remembered being driven to the doctors the next day to receive the missed needle.

Participants stated that even though they ‘believe in [vaccines]’, they generally do not know much about vaccines. Some thought there was ‘a lack of information’ given about why children receive certain immunizations at certain times. Young adults cited, ‘you get [a vaccine] for this and [a vaccine] for that’ but they do not really know why particular vaccines are given. In spite of this low knowledge, most participants still supported childhood vaccination.

*Parent 1*: ‘But to be honest I never knew that, that was what [HPV vaccination] was for. I just assumed it was for something—’

*Parent 2*: ‘Yeah they just go vaccinating—[laughter]—Just get it done!’ (Parents Table 2: First Question Round)

#### 3.2.2 Trust in Schools

As highlighted above, our discussions demonstrated that participants had low knowledge about school vaccines and vaccines generally. In light of this, we sought to understand why a parent in the Macedonian community would ‘just sign’ a school vaccine consent form. Participants of all ages reasoned that their community ‘trusts' schools and any information sent home. Parents had ‘faith in the system’ and thought schools look after children so, ‘of course we trust schools’. Parents agreed that the older generation (the first generation of migrants) always trusted schools and if a school sends home a notice about vaccines then children get the vaccine.

It's a must, it's a must, it's from school, so it's a must. (Parents Table 2: Third Question Round)

When we asked young adults about this, a few reasoned that because school vaccines are free, delivered through school, and seen to benefit children, older Macedonians would not question the school information.

[Parents] think it's safe because it's coming from the school. (Young Adults Table 1: Third Question Round)

Some young adults joked that, if their parents had to pay for school vaccines, ‘Then they'll ask the questions’.

In contrast to the majority, a small number of parents did not trust any information about vaccines. Based on their personal experiences with vaccine side‐effects, they lacked trust in government information and some thought ‘if [someone's] immune system is down, is slow, and it's weak’ vaccines could give a person ‘the sickness’ it is trying to prevent. When the tables were reshuffled, our facilitators brought up these ideas and comments to other parents. Many tended to disagree with these viewpoints or countered with their own more positive vaccine experiences and beliefs.

#### 3.2.3 Family Views are Passed on

Many participants remembered and discussed following their parents' choices regarding vaccination. Young adults thought that children trust their parents and that parents would not intentionally ‘mislead’ children about vaccines. Some thought it important for parents and the older generation to properly understand vaccine information since young people learn from their family, especially older family members.

…if you've got a good support system around you that's encouraging—um—engaging around vaccination then you'd be more inclined to agree with doing it. But if you have a parent or sibling or something that's dead against getting vaccinated, you'd be inclined to kind of think the same way. (Young Adult Table 1: Third Question Round)

And not just speak to the kids about it because like the parents is where we trust, and like even our grandparents, we absorb so much information from them and knowledge that—if they don't trust it—it comes down to us and we're like ‘Well if our parents don't trust it and our family doesn't trust it why would we trust it?’ You know what I mean? (Young Adults Table 1: Fourth Question Round)

Young adults provided us with examples of how differing family views can cause tension about who or what you trust (or mistrust) in regard to health matters. For one young adult, his family's distrust of medical professionals made him question when you would or would not see a doctor for a health matter. Hearing this, facilitators again asked why Macedonian parents would ‘just sign’ a school vaccine consent form. Reasoning always circled back to the trust parents place in the school.

#### 3.2.4 Impact of More HPV Vaccination Knowledge

Upon learning more about the school vaccination programme at the start of the evening, our participants discussed their new knowledge with each other and shared positive opinions about HPV vaccination. Young adults suggested that improving student knowledge about vaccines (i.e., ingredients, safety and benefits) in a student assembly could help reduce student anxiety or fear of needles. Some reasoned that this type of knowledge could also improve the conversations about vaccines students have within their peer groups.

And I know we've heard what meningococcal is, but we don't know exactly what happens if you get it. Sit us down, slide shows, all that. That would be helpful. (Young Adults Table 2: First Question Round)

Although young adults wanted their parents to be better informed about HPV vaccination, a few wondered whether more HPV vaccine knowledge could make their parents' vaccine‐hesitant.

But it's still like—if [parents] knew what [the vaccine] was they would be much more resistant I think to it. (Young Adult Table 1: Third Question Round)

This idea contrasted with the views of the parents, who agreed that most community members would support HPV vaccination and should be better informed about vaccine benefits. Some parents even wished the vaccine had been available ‘in my time’. Participants in both discussion groups also talked about whether HPV vaccination should be given in elementary school, saying that times are different now and that some children could be sexually active before Year 7. Parents were quick to qualify that they weren't referring to their own children, but children in general.

Today, a lot of young kids are having sex before 13. Too early, so I reckon this is too late, they should start it in the schools in primary. Before high school because it's getting worse now … they are all sexually transmitting … Early! (Parents Table 2: Third Question Round)

#### 3.2.5 Importance of Tailored Vaccine Communication

Participants were all supportive of their community having access to more information about HPV and school vaccination and this being provided in more diverse ways.

Parents supported school phone apps for distributing vaccination information and consent forms, as well as multiple media methods like Macedonian TV, radio programmes and magazines/newsletters. Young adults suggested emails and/or mailing information home could also improve parent access to vaccine information.

*Parent 1*: ‘Ours is called a [App Name]. So, everything goes into that School Bag App and now it's called [App Name]’.

*Researcher*: ‘And that tells you more information?’

*Parent 1*: ‘Yeah, that tells me everything’. (Parent, Table 3: First Question Round)

Many participants agreed that a multilingual, face‐to‐face information night or consultation would also be helpful for their community. An interactive event would allow community members to ask their own questions, in their own language and could provide ‘peace of mind’ regarding vaccine side effects. Young adults reasoned that translated information provided face‐to‐face may lead to parents and children taking the vaccine ‘more seriously’ and would demonstrate the importance of the health topic. When asked about translating information into Macedonian, participants agreed that most in the community know English now and language barriers are less common. Regardless, a few participants hinted that some community members would not have enough English skills to understand the more technical school vaccine information currently handed out.

I can understand it. But some things I can't. That's why I always pass the piece of paper or the information to my husband to be [clear]…. Like I know how to read but some words like—I'm thinking—geez what's this word. I have no idea. (Parent Table 1: Second Question Round)

## 4 DISCUSSION

This case study highlights the value of partnering with cultural navigators to engage with one local Australian‐Macedonian community in a community dialogue about HPV and school vaccination decisions and behaviours. For this community, school‐based vaccination behaviours were built on multilayer trust relationships and processes. This included historical and shared intergenerational attitudes, beliefs and experiences around vaccines and general community trust in schools. As school vaccination was experienced by some in Macedonia and is well established in NSW (Australia),^48^ HPV vaccination was seen as an extension of other childhood vaccines. HPV vaccination then benefits from community members' historically positive vaccine attitudes, beliefs and behaviours and is less reliant on parents and children fully understanding vaccine information. The Café method allowed participants to recognize and discuss what they did (and did not) know about vaccines and offer their own solutions for how HPV and school vaccine information could be improved for their community. Our research findings resonate with previous Australian literature linking trust in schools to parent's vaccine decision‐making^49^ and by applying the work of Larson et al.,^46^, ^47^ we further explore this multilevel trust relationship.

In Larson et al.'s ‘trust’ triangle, vaccination trust is made up of ‘trust in product’ (vaccine), ‘trust in provider’ (healthcare worker administering vaccines) and ‘trust in policy‐makers or institutions’ (health system, government or researchers) who approve and recommend vaccines. Our results slightly modify Larson et al.'s^46^ trust triangle (Figure 1) as the community saw schools as both vaccine ‘provider/administrator’ and an ‘institutional source recommending’ vaccines. This combination reinforces the ‘trust’ of the ‘vaccine product’. Larson et al.^46^ further explain that three types of external levers comprising of ‘generalised trust’ (general willingness to trust information), ‘historical trust’ (previous experiences) and ‘external influencers’ (trust in nonofficial sources of information) influence this trust triangle. In keeping with these levers, most of our participants demonstrated a ‘general willingness’ to trust all the information schools send home even without fully understanding the vaccine information. This ‘general willingness to trust’ combined with the lever of positive historical experiences of participating in other childhood/school vaccination programmes in both Macedonia and Australia, builds again on community trust in school vaccines. As younger members of the community trusted and stood by the decision‐making of their parents and older family members, the family can be seen as another ‘external influence’ trust lever. This explains why young people thought it important for parents and older family members to engage with and understand more about vaccines, as, in this community, family members have a strong role in teaching and guiding the younger generation.

During Cafés, our participants also identified that they want to engage with tailored and translated health information in ‘multi‐modal’ ways (i.e., information nights, media sources) and from trusted sources. This tailoring and translating not only makes the information understandable but demonstrates that the health topic is worth engaging with. These are important findings, as much work on promoting vaccine uptake in minority populations focuses primarily on improving vaccine information, knowledge or reminder systems^50^ rather than tailoring health information to come from trusted sources or building ‘public trust’ in the vaccine. By working collaboratively with health departments that share similar health goals (i.e., HPV cancer prevention) and are already embedded and trusted within CALD communities, school immunization providers could leverage these relationships to improve and tailor key vaccination messages.^20^

### 4.1 Strengths and limitations

This case study has a number of strengths. First, by applying multiple analytical frameworks^44^, ^45^, ^46^, ^47^ to draw out trust processes within one CALD community, we show how HPV vaccination interventions could build on and strengthen community motivations to vaccinate. Our research also occurred as a place‐based study using a participatory research method of the World Café—so insights were gained using active communication.^27^ Though not generalizable to other CALD communities, investigative methods and frameworks are transferable to other communities.

Limitations include conducting Cafes at a single site, the small number of participants and the lack of engagement with fathers. Also, family dyads had attended each Café (i.e., mothers and daughters or mothers and sons), but we were unable to identify who or how many made up these family groups. Future research could adapt these methods to collect information regarding participating family groups and be more engaging for male parents.

## 5 CONCLUSION

By partnering with trusted cultural navigators and using participatory engagement methods such as World Cafes, this study highlights the value research partnerships bring when investigating vaccine motivations in individual CALD communities. The use of multiple theoretical frameworks also offers a fresh perspective on how aligning a vaccination programme with a trusted institution (the school) contributes to one CALD community's vaccine engagement and behaviour with HPV and school vaccination. To support vaccination interventions in communities where vaccination mistrust may exist,^51^ future research could expand community health partnerships to include cultural navigators from trusted charitable or religious groups,^52^ trusted community health workers^12^, ^53^ and/or trusted media sources.^54^

## AUTHOR CONTRIBUTIONS

*Conception, supervision*: Kathleen Prokopovich, Lyn Phillipson, Annette Braunack‐Mayer and Leissa West (Pitts). *Study design*: Kathleen Prokopovich, Lyn Phillipson, Annette Braunack‐Mayer and Jackie Street. *Study coordination*: Kathleen Prokopovich. *Recruitment and language support*: Biljana Stanoevska. *Facilitation*: Lyn Phillipson and Jackie Street. *Preliminary data analysis and original manuscript draft*: Kathleen Prokopovich. *Data interpretation*: Lyn Phillipson, Annette Braunack‐Mayer and Biljana Stanoevska. All authors contributed to the final edits of the manuscript. All authors attest they meet the ICMJE criteria for authorship.

## CONFLICT OF INTEREST

The authors declare no conflict of interest.

## ETHICS STATEMENT

This study received ethical approval by the University of Wollongong/Illawarra‐Shoalhaven Local Health District Joint Health & Medical Human Research Ethics Committee (2019/ETH12648).

## Supporting information

Supplementary information.

## Data Availability

Data are available on request due to privacy/ethical restrictions. The data that support the findings of this study are available upon reasonable request from the corresponding author. The data are not publicly available due to privacy or ethical restrictions.
