# Supplementary material for: Using World Cafés to engage an Australian culturally and linguistically diverse community around human papillomavirus vaccination
Source: Health Expect. 2023 Feb 16;26(3):1039–51. doi: 10.1111/hex.13703 (PMC10154861; doi:10.1111/hex.13703)
Supplement: Supplementary file 1 — Supplementary information. [file HEX-26--s001.pdf]

SUPPLIMENTARY MATERIALS FILE

Table 1. World Café Program

Time (mins)	Activity	Participant Response
**20 mins**	The first author introduced herself, the timetable for the evening and gave a brief presentation about the school-based vaccination program & HPV vaccination.	Participants were able to ask any questions about vaccination and were invited to share what comes to mind when they think of a vaccine.
	Table Questions:<br>*Table 1: What do you think about high school students receiving vaccinations at school? What does your community think?*<br><br>*Table 2: What do you think about the human papillomavirus vaccination? What do members of your community think?*<br><br>*Table 3: For you and other people from your community, what is the best way to give information about and get consent for the human papillomavirus vaccination?*	
**15 mins**	Questions Session 1: Discussions at each table.	Research Hosts stay at the same table throughout. Throughout the process hosts write notes of key points on the white table paper as people talk. Other participants are encouraged to do the same. Everyone else changes tables after each session.
**5 mins**	Break and change tables	
**10 mins**	Question Session 2: Discussion at each table.	Research Hosts give brief explanation of the key points raised by the last group. Hosts stay at the same table. Everyone else changes tables.
**5 mins**	Break and change tables	
**10 mins**	Question Session 3: Discussion at each table.	Hosts give brief explanation of the key points raised by the last group. Hosts stay at the same table. Everyone else changes tables.
**10 mins**	Break and change tables: *Fourth Question: From your perspective, if the high school vaccination program was run differently, what would make it better for you and your community members?*	
**10-15 mins**	Session 4: Discussion at each table.	Hosts give brief explanation of the key points raised by the last group. Hosts stay at the same table and repeat 4th question.

**5mins**	Break and change tables- Repeat 4th Question*	
**5-10 mins**	Session 4: Discussion at each table.*	Hosts give brief explanation of the key points raised by the last group. Hosts stay at the same table and repeat 4th question. * This session may be cut short if it seems participants are restless/tired of discussion.
**20 mins**	Longer break – during break the Researcher Hosts bring table papers to the first author. The first author summarised 6-8 main points from each question onto PowerPoint slides for sharing with the group.	
**15-20 mins**	The first author presented the Powerpoint slides with the participants and facilitated a general discussion about the insights.	Participants clarified any points and pointed out what they strongly agreed with.
**Finish**		

* Only occurred in the first World Café event.

Table 2. Summary of Table Discussion Guides

**Table Number**	**Question**	**Prompts**
All Tables	*What word or words come to mind when you think of a vaccine?*	[Table host can write down what they think first]
Table 1	*What do you think about high school students receiving vaccinations at school? What does your community think?*	• Why could a vaccination program work in a high school? Does this program make it easier or harder to get a vaccine? How so?<br>• So if we think back to the presentation, students get vaccinated if they bring back a signed consent form before they get vaccinated. Do you think this process works? Why or why not?<br>• What thoughts/feelings come to mind when you think of a school being used for vaccinating teenagers?<br>• Do you like/not like the idea of students being vaccinated at school? Why or why not?<br>• Who agrees with what has been said? Why do you agree?<br>• What if someone in your community had a different view? Why do you think they could hold different views?
Table 2:	*What do you think about the human papillomavirus vaccination? What do*	• How could the HPV vaccine help someone?<br>• Who might not be helped by the HPV vaccine?

	members of your community think?	• Who agrees with what has been said and why?<br>• Could someone have a different thought about what's being said here? What would they say? What reasons would someone have to think this way?
Table 3	*For you and other people from your community, what is the best way to give information about and get consent for the human papillomavirus vaccination?*	• How much information is needed for parents to consent? What information do you think they need?<br>• What about students? What information would a student need?<br>• Where could parents and students get this information?<br>• Who should give this information to parents or students?<br>• What did you know about the school vaccination program before today?
All tables	*From your perspective, if the high school vaccination program was run differently, what would make it better for you and your community members?*	• What could be done to make people think that the high school vaccination program is important? Who could help with this?<br>• What could make a parent not consent for their child to participate in the high school vaccination program? What could be done to change this?<br>• What could make a student not want to participate in the high school vaccination program? What could be done to change this?<br>• What could make parents or students more comfortable about being vaccinated in a school?

Table 3. Example of deductive and inductive themes for data analysis.

**Theme**	**Subthemes**	**Sample Quotes**
We've always vaccinated	<u>Deductive (TIP Framework)</u><br>Vaccine knowledge (Capability).<br>Vaccines protect/help (Motivation).<br>Children always vaccinated (Social Opportunity & Motivation).<br><br><u>Inductive (Narrative Inquiry)</u><br>Motivation to understand vaccine information (Social & Emotional context of vaccination).<br>Relationship of past experiences to future decisions (Temporality).	*"I think when [parents] first have their kids they [have] an understanding what vaccinations are- they can get it from 6 months, 4 years, it's something that is always done for their kids. So, I guess looking into the understanding of what these new vaccinations are they don't really care necessarily. They just know it's going to help their children."*<br>***-Young adults table 1: Second question period***

		Parent 1*: "But to be honest I never knew that, that was what [HPV vaccination] was for. I just assumed it was for something-"*<br>***Parent 2****: "Yeah they just go vaccinating-[laughter]-Just get it done!"*<br>***-Parents Table 2: First Question Round***
We Trust the Schools	<u>Deductive (TIP Framework)</u><br>Vaccine acceptance (Motivation).<br>Vaccination attitudes (Motivation).<br>Accessing vaccines (Opportunity-Physical).<br>Parents normally follow school recommendations (Opportunity-Social).<br><br><u>Inductive (Narrative Inquiry)</u><br>Schools as a location (Place).<br>Schools are safe (Social & Emotional context of vaccination).<br>Personal/community motivations to trust information source (Social & Emotional context of vaccination).	*"It's a must, it's a must, it's from school, so it's a must."*<br>***- Parents Table 2: Third Question Round***<br><br>*"[Parents] think it's safe because its coming from the school."*<br>***-Young Adults Table 1: Third Question Round***
Family Views Are Passed Down	<u>Deductive (TIP Framework)</u><br>Family support (Opportunity-Social).<br>Vaccine Attitudes/Beliefs (Motivation).<br><br><u>Inductive (Narrative Inquiry)</u><br>Relationship between family vaccine motivations to individual motivations (Social & Emotional context of vaccination).<br>Historical cultural customs (Temporality; Social & Emotional context of vaccination).	*"…if you've got a good support system around you that's encouraging-um-engaging around vaccination then you'd be more inclined to agree with doing it. But if you have a parent or sibling or something that's dead against getting vaccinated, you'd be inclined to kind of think the same way."*<br>***-Young Adult Table 1: Third Question Round***<br><br>*"And not just speak to the kids about it because like the parents is where we trust, and like even our grandparents, we absorb so much information from them and knowledge that- if they don't trust it- it comes down to us and we're like "Well if our parents don't trust it and our family doesn't trust it why would we trust it?" You know what I mean?"*<br>**-Young Adults Table 1: Fourth Question Round**
Impacts of Increasing HPV vaccination knowledge.	<u>Deductive (TIP Framework)</u><br>Vaccine Knowledge (Capability).<br>Access/understanding vaccine information (Opportunity-Physical).<br><br><u>Inductive (Narrative Inquiry)</u><br>Child's perception of parents (Social & Emotional context of vaccination).	*"But it's still like- if they [parents] knew what it [the vaccine] was they would be much more resistant I think to it.."*<br>***-Young Adult Table 1: Third Question Round***<br><br>*"And I know we've heard what meningococcal is, but we don't know*

	Relationship between knowledge (Capability) and perceived risk of disease (Motivations) (Social & Emotional context of vaccination).	*exactly what happens if you get it. Sit us down, slide shows, all that. That would be helpful."*<br>***- Table 2, First Question Round***<br><br>*"Today, a lot of young kids are having sex before 13. Too early, so I reckon this is too late, they should start it in the schools in primary. Before high school because it's getting worse now…they are all sexually transmitting...Early!"*<br>***-Parents Table 2: Third Question Round***
Tailored Vaccination Communic ation.	<u>Deductive (TIP Framework)</u><br>Vaccine knowledge (Capability)<br>Understandable information (Capability & Opportunity- Physical).<br>Communication methods (Opportunity-Physical).<br><br><u>Inductive (Narrative Inquiry)</u><br>Relationship between vaccine literacy (Capability), vaccine confidence (Motivation) and preferred communication methods (Opportunity-Physical) (Social & Emotional context of vaccination).<br>Schools as a learning space (Place).	***Parent 1****: "Ours is called a [App Name]. So, everything goes into that School Bag App and now it's called [App Name]. "*<br>***Researcher:*** *"And that tells you more information?"*<br>***Parent 1:*** *"Yeah, that tells me everything."*<br>***- Parent, Table 3: First Question Round***<br><br>*"I can understand it. But some things I can't. That's why I always pass them piece of paper or the information to my husband to be [clear]…. Like I know how to read but some words like-I'm thinking-geez what's this word. I have no idea."*<br>- ***Parent, Table 1: Second Question Round***

THE SCHOOL-BASED VACCINATION PROGRAM: A COMMUNITY DISCUSSION

A UNIVERSITY OF WOLLONGONG & ISLHD-MULTICULTURAL HEALTH SERVICE PARTNERSHIP

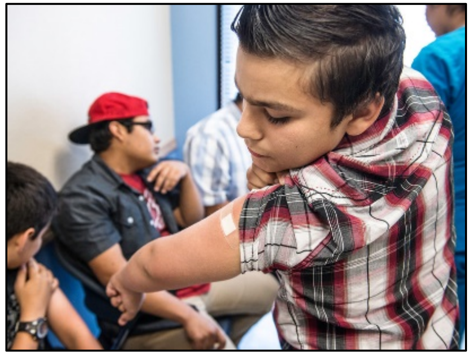

UNIVERSITY OF WOLLONGONG AUSTRALIA

NSW GOVERNMENT | Health | Illawarra Shoalhaven Local Health District

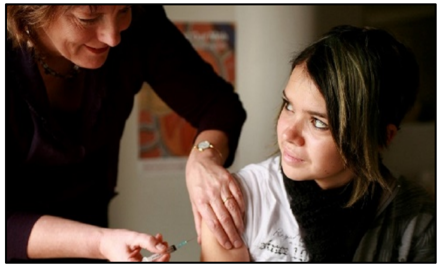

ДОБРЕДОЈДОВТЕ

Housekeeping:

- Toilets
- Emergency Exits
- Leave at 8:30pm ☺

EVENING SCHEDULE

- Short Presentation (25 minutes)
- Discuss 4 questions with 5 minute short breaks in between (60 minutes)
- Longer break (15 minutes)
- Final Discussion (10 minutes)
- Get up at any time ☺

THE NSW SCHOOL VACCINATION PROGRAM…….
WHY TALK ABOUT IT?

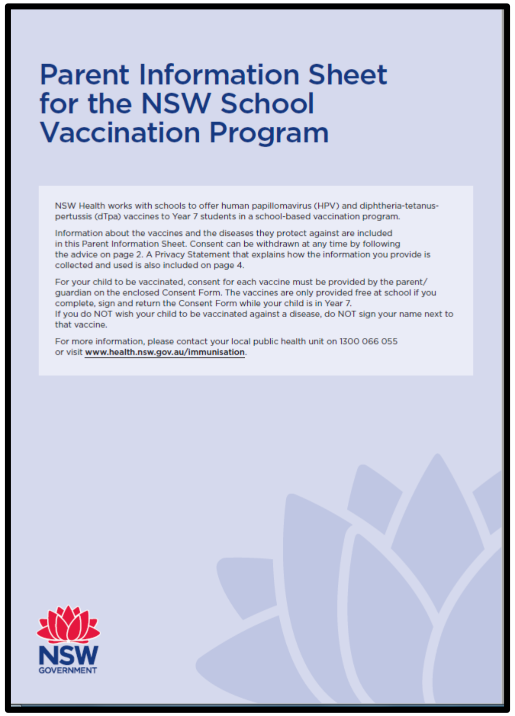

Parent Information Sheet for the NSW School Vaccination Program

NSW Health works with schools to offer human papillomavirus (HPV) and diphtheria-tetanus-pertussis (dTpa) vaccines to Year 7 students in a school-based vaccination program.

Information about the vaccines and the diseases they protect against are included in this Parent Information Sheet. Consent can be withdrawn at any time by following the advice on page 2. A Privacy Statement that explains how the information you provide is collected and used is also included on page 4.

For your child to be vaccinated, consent for each vaccine must be provided by the parent/guardian on the enclosed Consent Form. The vaccines are only provided free at school if you complete, sign and return the Consent Form while your child is in Year 7.
If you do NOT wish your child to be vaccinated against a disease, do NOT sign your name next to that vaccine.

For more information, please contact your local public health unit on 1300 066 055 or visit www.health.nsw.gov.au/immunisation.

NSW GOVERNMENT

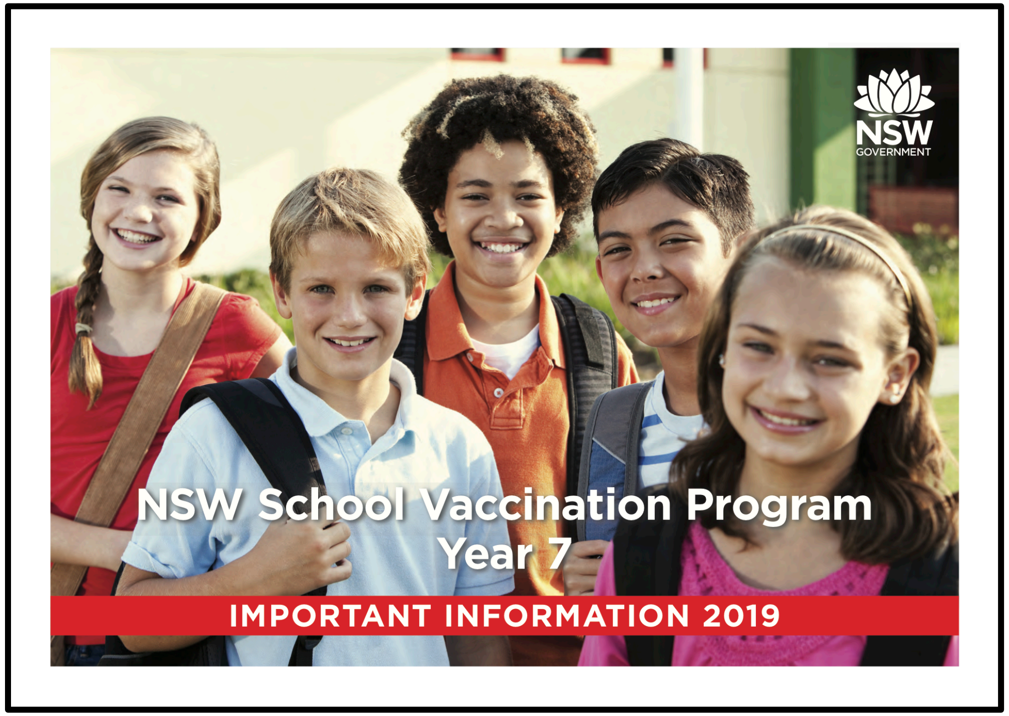

Parent Information Sheet NSW School Vaccination Program: https://www.health.nsw.gov.au/immunisation/Publications/year-7.pdf

WHAT IS A VACCINE?

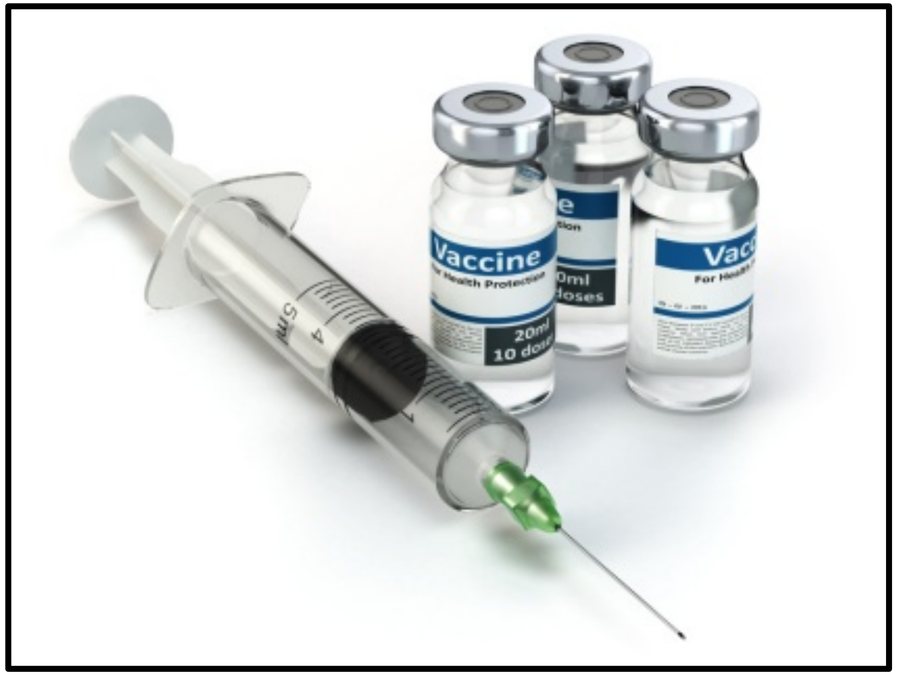

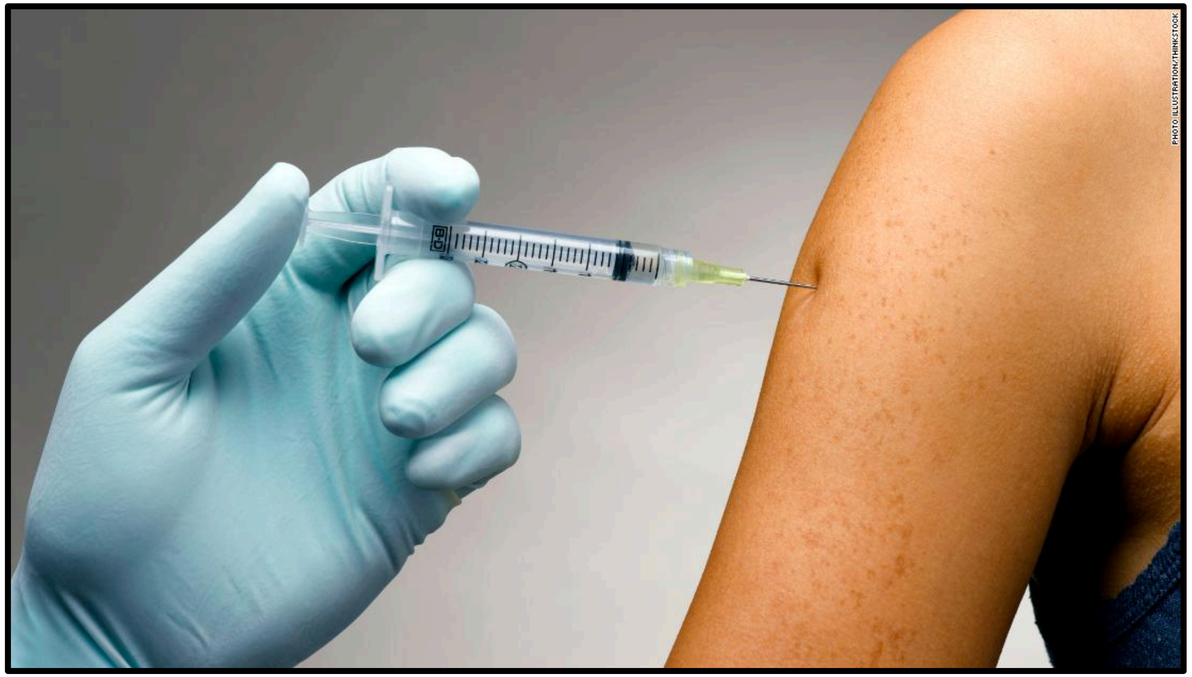

Vaccine Photo Credit 1: https://www.aafp.org/news/health-of-the-public/20180620vaccineexempts.html

NATIONAL IMMUNISATION PROGRAM (NIP)

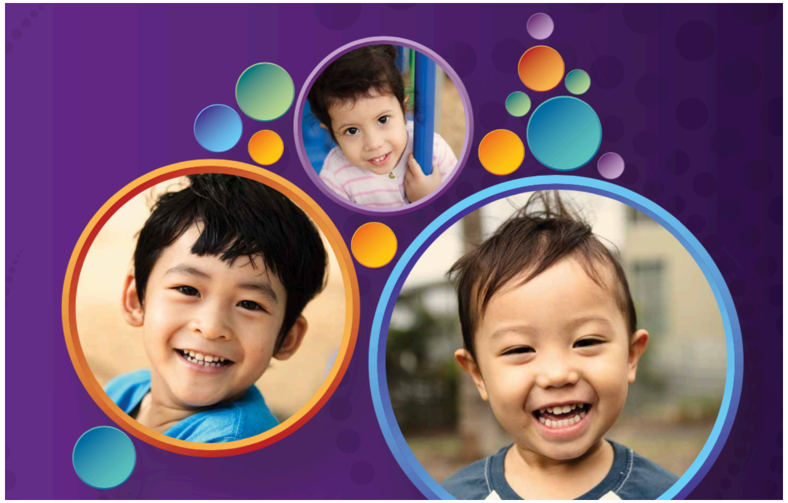

https://www.health.gov.au/health-topics/immunisation

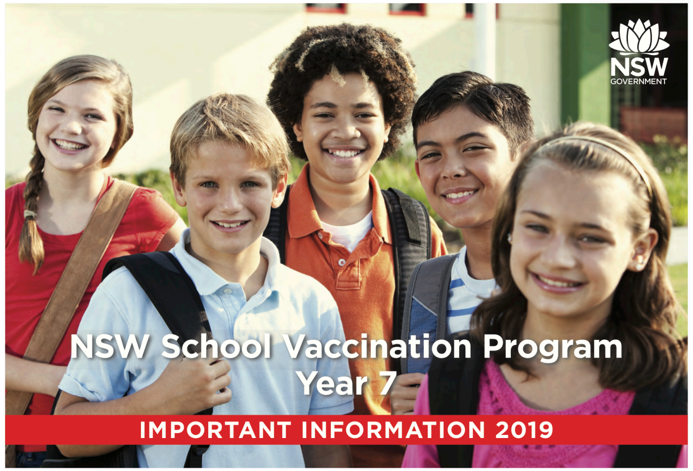

https://www.health.nsw.gov.au/immunisation/Publications/year-7.pdf

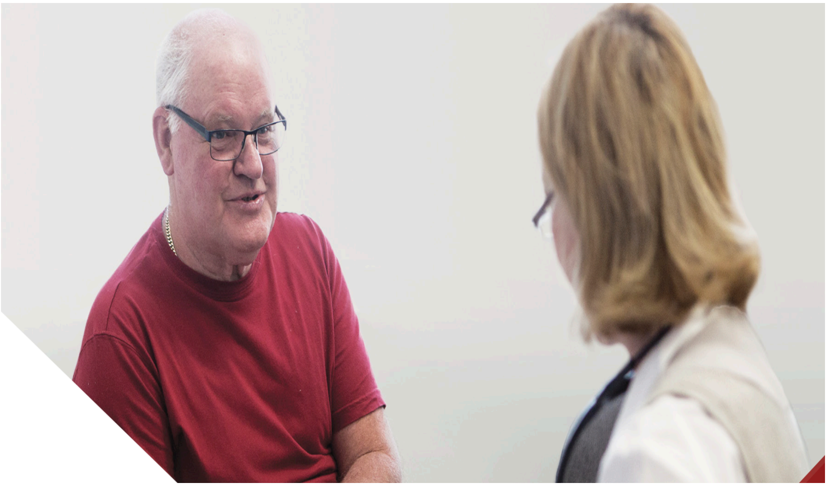

https://www.health.gov.au/sites/default/files/resources/images/shingles-poster.pdf

THE NSW SCHOOL VACCINATION PROGRAM

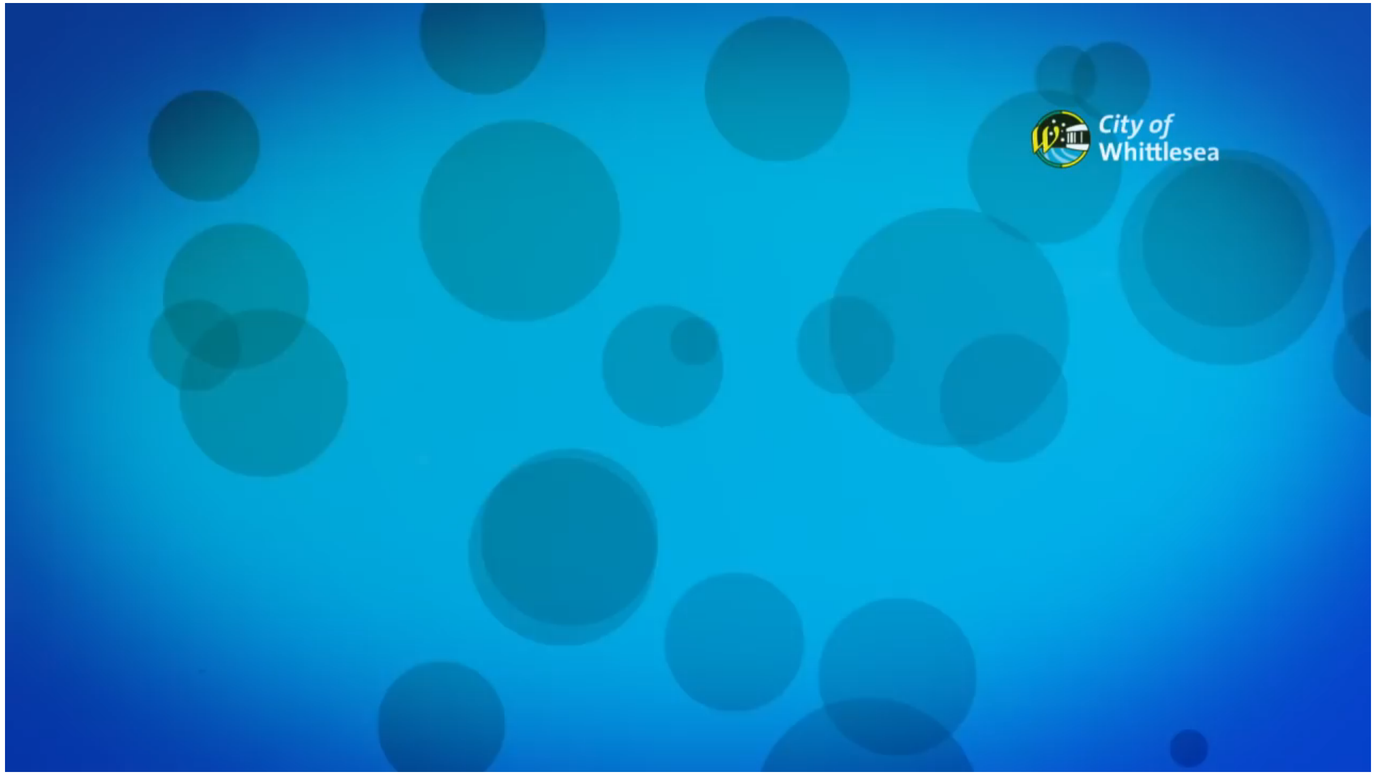

https://youtu.be/ynadF2I7w6I

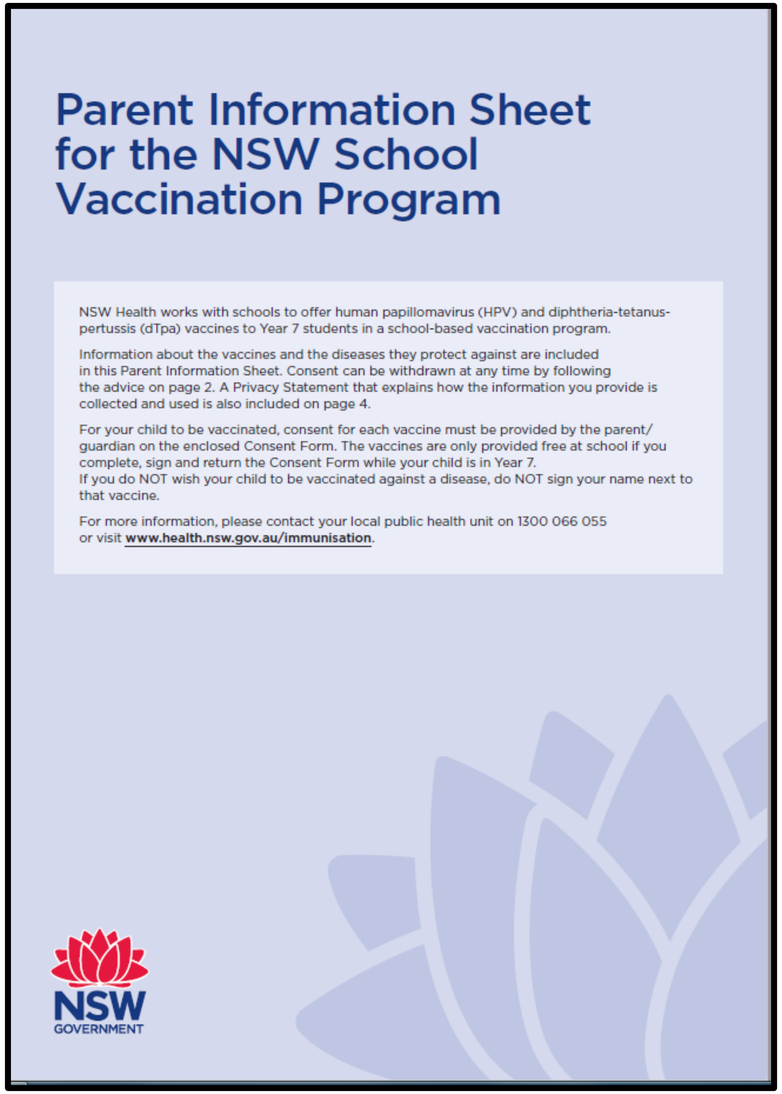

Parent Information Sheet for the NSW School Vaccination Program

NSW Health works with schools to offer human papillomavirus (HPV) and diphtheria-tetanus-pertussis (dTpa) vaccines to Year 7 students in a school-based vaccination program.

Information about the vaccines and the diseases they protect against are included in this Parent Information Sheet. Consent can be withdrawn at any time by following the advice on page 2. A Privacy Statement that explains how the information you provide is collected and used is also included on page 4.

For your child to be vaccinated, consent for each vaccine must be provided by the parent/guardian on the enclosed Consent Form. The vaccines are only provided free at school if you complete, sign and return the Consent Form while your child is in Year 7.
If you do NOT wish your child to be vaccinated against a disease, do NOT sign your name next to that vaccine.

For more information, please contact your local public health unit on 1300 066 055 or visit www.health.nsw.gov.au/immunisation.

NSW GOVERNMENT

Parent Information Sheet NSW School Vaccination Program: https://www.health.nsw.gov.au/immunisation/Publications/year-7.pdf
HPV Vaccination For Teens, School-immunisation Still Shot: https://www.youtube.com/watch?time_continue=38&v=ynadF2I7w6I

WHY IS THE HUMAN PAPILLOMAVIRUS (HPV) VACCINE GIVEN?

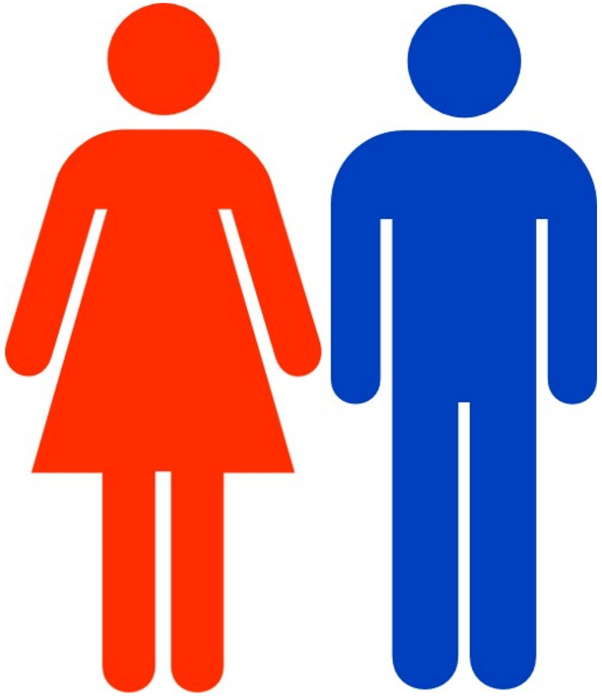

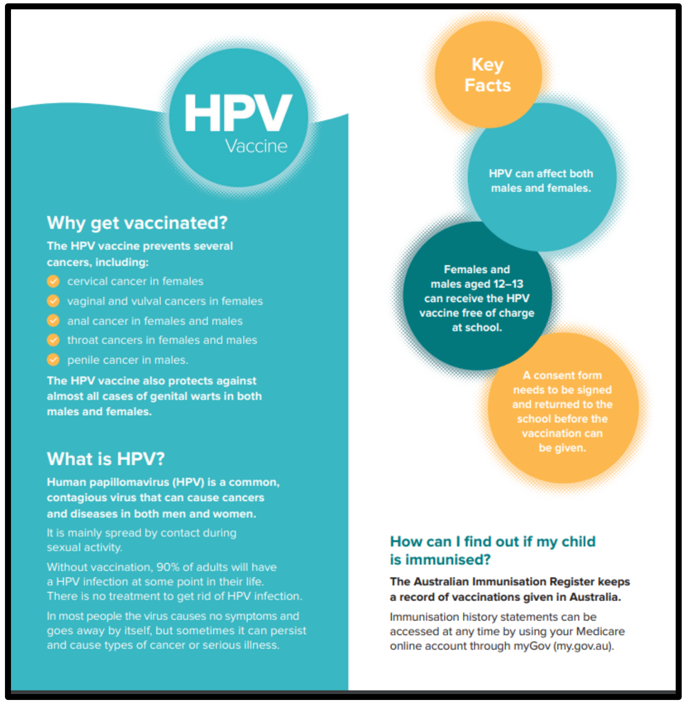
HPV
Vaccine

Why get vaccinated?

The HPV vaccine prevents several cancers, including:
- cervical cancer in females
- vaginal and vulval cancers in females
- anal cancer in females and males
- throat cancers in females and males
- penile cancer in males.

The HPV vaccine also protects against almost all cases of genital warts in both males and females.

What is HPV?

Human papillomavirus (HPV) is a common, contagious virus that can cause cancers and diseases in both men and women.

It is mainly spread by contact during sexual activity.

Without vaccination, 90% of adults will have a HPV infection at some point in their life. There is no treatment to get rid of HPV infection.

In most people the virus causes no symptoms and goes away by itself, but sometimes it can persist and cause types of cancer or serious illness.

Key Facts

HPV can affect both males and females.

Females and males aged 12–13 can receive the HPV vaccine free of charge at school.

A consent form needs to be signed and returned to the school before the vaccination can be given.

How can I find out if my child is immunised?

The Australian Immunisation Register keeps a record of vaccinations given in Australia.

Immunisation history statements can be accessed at any time by using your Medicare online account through myGov (my.gov.au).

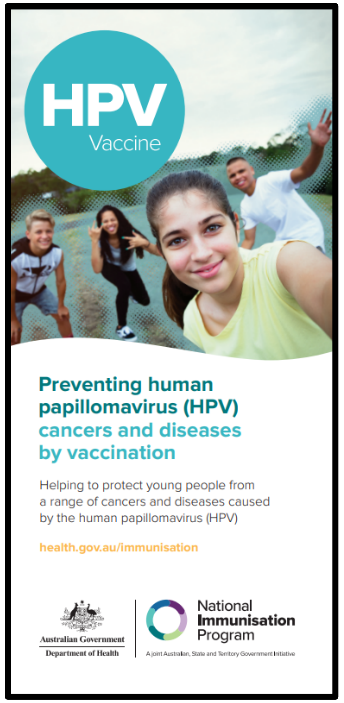
HPV
Vaccine

Preventing human papillomavirus (HPV) cancers and diseases by vaccination

Helping to protect young people from a range of cancers and diseases caused by the human papillomavirus (HPV)

health.gov.au/immunisation

Australian Government Department of Health

National Immunisation Program

Information in: HPV Vaccine Brochure for parents: https://www.health.gov.au/sites/default/files/preventing-hpv-cancers-and-diseases-by-vaccination-brochure.pdf

HOW THIS COMMUNITY DISCUSSION WILL WORK:

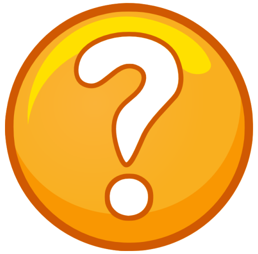

- You will have a chance to answer 4 questions;
- Each table has a different question;

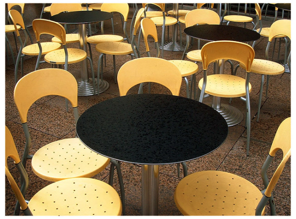

- To answer each question, you have to get up and move tables;
- You will have 10-15 minutes to discuss each question

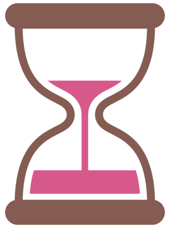

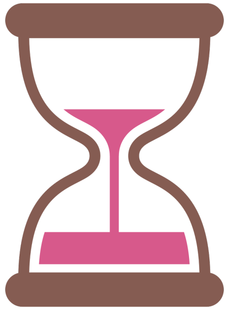

(5 MINUTES)

What word(s) come to mind when you think of a vaccine?

Introduce yourself and answer

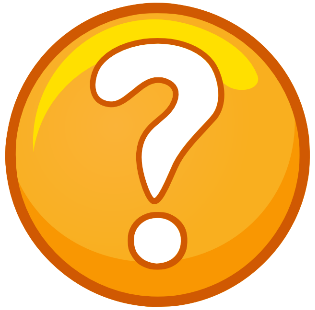

(15 minutes to discuss)

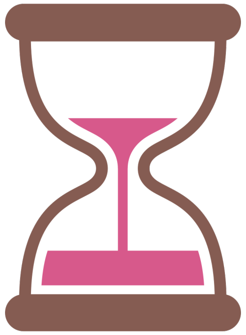

Things to think about at your table:

- What thoughts or feelings come to mind?
- What could someone from your community think?
- Could someone have a different view? What view could they have?

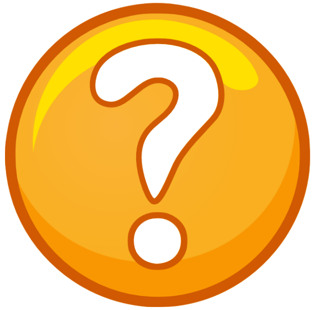

Find a new table (10 minutes to discuss)

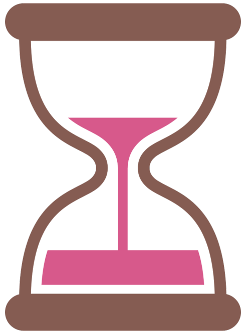

Things to think about at your table :

- What thoughts or feelings come to mind?
- What could someone from your community think?
- Could someone have a different view? What view could they have?

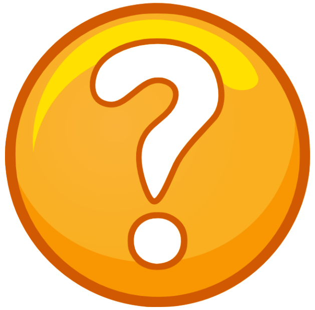

Find a new table
(5 minutes to discuss)

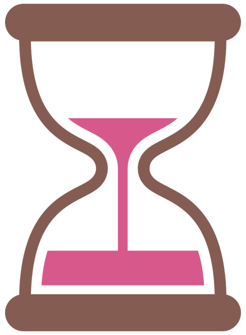

Things to think about at your table :

- What thoughts or feelings come to mind?
- What could someone from your community think?
- Could someone have a different view? What view could they have?

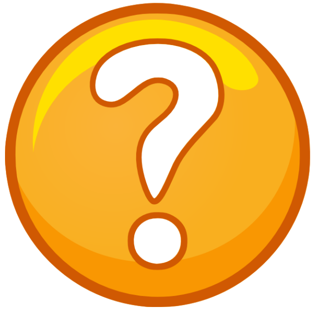

Find a new table
(5 minutes to discuss)

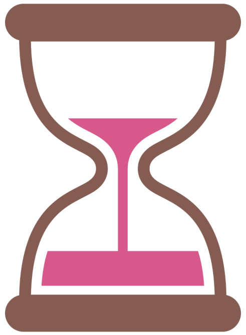

QUESTION FOR EVERY TABLE:

If the high school vaccination program was run differently what would make it better for you and your community members?

LETS TAKE A BREAK

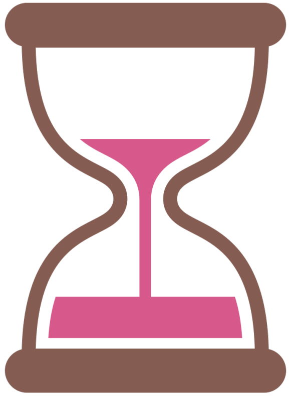

Break (10-15 minutes)

SUMMARY QUESTION 1: SCHOOL USED AS A VACCINE CLINIC

- It must be good if its from the school (good place to be vaccinated)
- Good that its free in schools
- School is a second home for some kids
- Consent process could be better (parents just sign but don't know or don't discuss what kids are getting)
- Felt information given is inadequate so could lead to mistrust
- Even though they speak English would like more Macedonian language information
- Parents and grandparents trust Macedonian Dr's since they speak the same language, information needs to be passed through the generations
- For parents, the school program is an acceptable setting to be vaccinated.

SUMMARY QUESTION 2: WHAT DO YOU THINK OF THE HPV VACCINE?

- Brand new information (even if they had gotten it in Year 7)
- Amazing that it could prevent cancer
- Good/positive attitude towards vaccine
- Want more information so they could talk to friends who are doubtful
- New information that it could help boys too
- Some people may think they were getting a vaccine for HIV not HPV (related to sexual transmission)

SUMMARY QUESTION 3: WHAT'S THE BEST WAY TO COMMUNICATE INFORMATION AND GET CONSENT?

- Electronic media (newsletter, app, email DIRECT TO PARENT)
- Information to both parent and student, but consent directly to parent
- Students don't know what they are getting, would like more information (class assembly→ talk about benefits)
- Grandparents are concerned about them and should know too
- Language not a big barrier since people can understand English even if they can't speak it

SUMMARY QUESTION 4: WHAT WOULD MAKE THE PROGRAM BETTER FOR ME AND MY COMMUNITY?

- More information in Macedonian language
- Formats that are appropriate for kids (multiple ways of giving the information)
- Go through kids, parents, grandparents (Social community clubs)
- Too technical right now (language)
- Promote benefits
- Information for parents and grandparents, & government (trust in school and health department)
- Record of what was given
- Information to how to counter negative vaccine attitudes
- Opt out of programme
- Sharing personal stories

ANYTHING ELSE?

Ви благодарам ☺

THANK YOU!

Kathleen Prokopovich: kar715@uowmail.edu.au

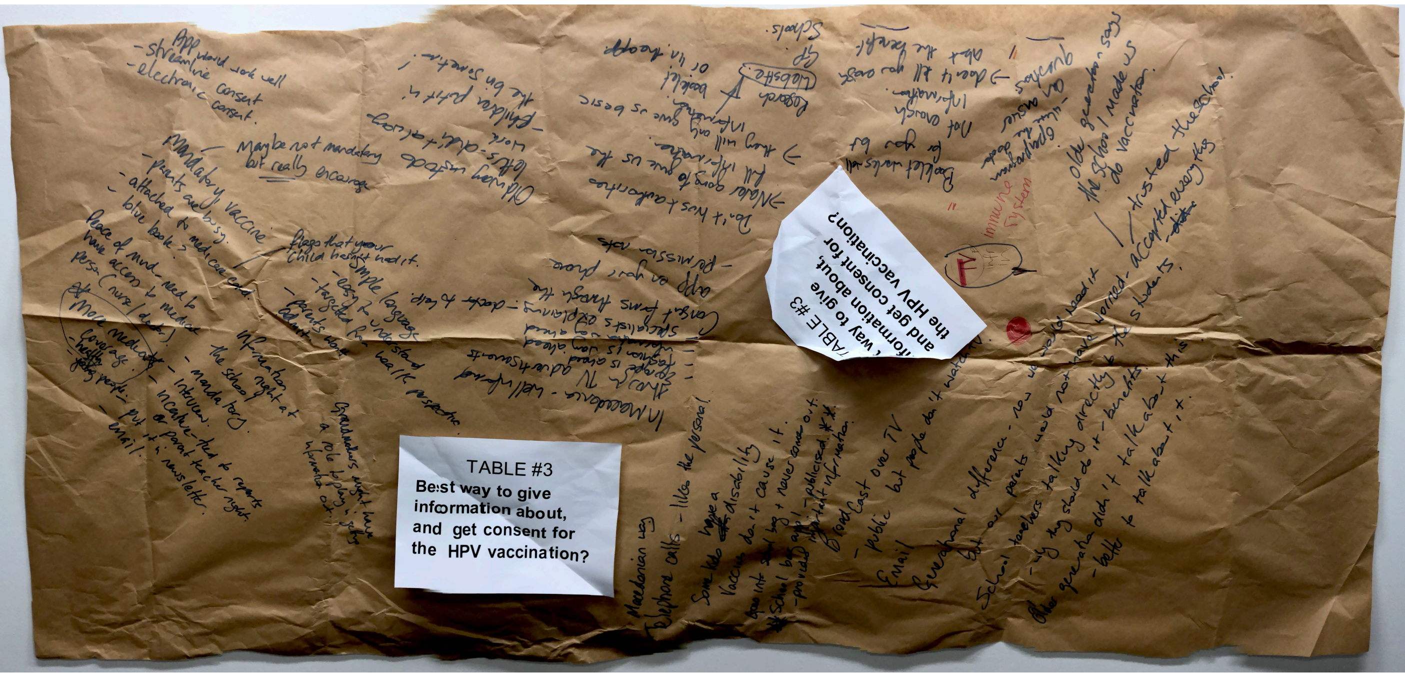
TABLE #3
Best way to give information about, and get consent for the HPV vaccination?
App would work well
- streamline consent
- electronic consent.
Maybe not mandatory but really encourage
Mandatory vaccine
Broadcast over TV
Email
